# Functional connectivity abnormalities in Type I Chiari: associations with cognition and pain

**DOI:** 10.1093/braincomms/fcab137

**Published:** 2021-06-14

**Authors:** Michelle L Houston, James R Houston, Ken Sakaie, Petra M Klinge, Sarel Vorster, Mark Luciano, Francis Loth, Philip A Allen

**Affiliations:** 1 Department of Psychology, The University of Akron, Akron, OH 44325, USA; 2 Department of Psychology, Middle Tennessee State University, Murfreesboro, TN 37132, USA; 3 Department of Diagnostic Radiology, The Cleveland Clinic, Cleveland, OH 44195, USA; 4 Department of Neurosurgery, Rhode Island Hospital, and Warren Alpert Medical School, Brown University, Providence, RI 02903, USA; 5 Department of Neurological Surgery, The Cleveland Clinic, Cleveland, OH 44195, USA; 6 Department of Neurosurgery, Johns Hopkins Medical Center, Baltimore, MD 21224, USA; 7 Department of Biomedical Engineering, The University of Akron, Akron, OH 44325, USA; 8 Department of Mechanical Engineering, The University of Akron, Akron, OH 44325, USA

**Keywords:** Chiari malformation, cerebellar disease, functional magnetic resonance imaging, brain microstructure, chronic pain

## Abstract

There is initial evidence of microstructural abnormalities in the fibre-tract pathways of the cerebellum and cerebrum of individuals diagnosed with Type I Chiari malformation. However, it is unclear whether abnormal white matter architecture and macro-level morphological deviations that have been observed in Chiari translate to differences in functional connectivity. Furthermore, common symptoms of Chiari include pain and cognitive deficits, but the relationship between these symptoms and functional connectivity has not been explored in this population. Eighteen Type I Chiari patients and 18 age-, sex- and education-matched controls underwent resting-state functional MRI to measure functional connectivity. Participants also completed a neuropsychological battery and completed self-report measures of chronic pain. Group differences in functional connectivity were identified. Subsequently, pathways of significant difference were re-analyzed after controlling for the effects of attention performance and self-reported chronic pain. Chiari patients exhibited functional hypoconnectivity between areas of the cerebellum and cerebrum. Controlling for attention eliminated all deficits with the exception of that from the posterior cerebellar pathway. Similarly, controlling for pain also eliminated deficits except for those from the posterior cerebellar pathway and vermis VII. Patterns of Chiari hyperconnectivity were also found between regions of the cerebellum and cerebrum in Chiari patients. Hyperconnectivity in all regions was eliminated after controlling for attention except between left lobule VIII and the left postcentral gyrus and between vermis IX and the precuneus. Similarly, hyperconnectivity was eliminated after controlling for pain except between the default mode network and globus pallidus, left lobule VIII and the left postcentral gyrus, and Vermis IX and the precuneus. Evidence of both hyper- and hypoconnectivity were identified in Chiari, which is posited to support the hypothesis that the effect of increased pain in Chiari draws on neural resources, requiring an upregulation in inhibitory control mechanisms and resulting in cognitive dysfunction. Areas of hypoconnectivity in Chiari patients also suggest disruption in functional pathways, and potential mechanisms are discussed.

## Teaser

Resting-state fMRI imaging was conducted on a group of Type I Chiari malformation patients and age- and education-matched controls. Analyses yielded patterns of relative hypo- and hyperconnectivity, associations between differences in functional connectivity and self-reported chronic pain, and relationships between differences in functional connectivity and attentional capacity.

## Graphical Abstract

**Graphical Abstract:**
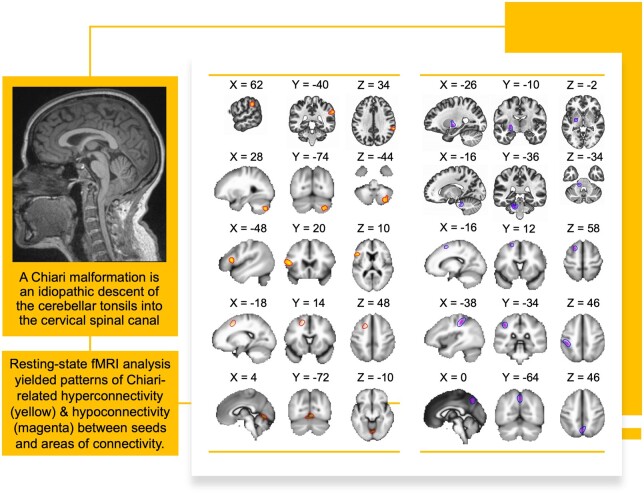

## Introduction

Type I Chiari malformation (CMI) is defined radiologically as the descension of the cerebellar tonsils by at least 5 mm below the foramen magnum.^1^ Common symptoms of CMI include headache and neck pain,^2^ sensory impairment including visual and balance issues,^3^ as well as cognitive dysfunction, especially related to attention, working memory and visuospatial reasoning.^4–7^ Neuropsychiatric symptoms, such as depression, anxiety, and affective dysregulation, have also been reported in CMI patients.^3^^,^^7^

There are two goals in this study. First, CMI-related differences in functional connectivity, as indexed by resting-state fMRI (rs-fMRI), are assessed using a case–control design. Second, the relationship between functional connectivity differences and symptom severity is examined by re-analyzing pathways of significant difference after covarying for cognitive performance and self-reported pain. To date, there have been two prior studies that have correlated CMI symptoms with neuroimaging indices associated with neural microarchitecture.^8^^,^^9^ These studies used diffusion tensor imaging (DTI) and provide evidence of differences in DTI signals between CMI patients and age- and education-matched controls, some of which were related to cognitive dysfunction and self-reported chronic pain.^8^ Whereas DTI is commonly believed to provide an index of the structural connectivity of neural white matter, rs-fMRI is thought to assess functional interconnectivity of brain regions.^10^

There is accumulating evidence that cognitive dysfunction is a common symptom of CMI.^4–7^^,^^11^^,^^12^ In previous studies, deficits in CMI were found in executive function,^4–7^^,^^11^ immediate recall,^11^^,^^13^ working memory^11^ and visuospatial skill.^11^ It was proposed that these deficits may be further exacerbated by comorbid affective disturbances (e.g. depression, anxiety, stress) and pain.^5^^,^^8^^,^^11^^,^^13^

It has also been demonstrated that the experience of chronic pain can divert cognitive resources away from the task at hand, causing a reduction in performance.^14^ Specific to CMI, Houston et al.^5^ identified CMI-related deficits in immediate memory, delayed memory and attention using a standardized neuropsychological assessment. However, after controlling for self-reported pain, only deficits in attention remained. Similarly, Allen et al.^4^ and García et al.^11^ observed poorer cognitive performances in CMI patients relative to controls that were at least partly associated with self-reported pain. Allen et al.^13^ provided a more nuanced investigation of the dynamics between cognitive function and pain in CMI. In this study, CMI patients with low, but not high, levels of self-reported pain exhibited cognitive benefits from engaging in self-focused reflection. Collectively, these results suggest that distraction due to pain is involved in cognitive deficits, but that this is not a complete explanation of cognitive dysfunction in CMI.

Using DTI, Kumar et al.^9^ and Houston et al.^8^ were the first to determine whether DTI measures were associated with cognitive function. Kumar et al. observed a negative correlation between visual attention and task switching as measured by the Trail Making Test and the DTI measure of fractional anisotropy^15^ (FA) in the genu as well as the splenium. Alternatively, Houston et al.^8^ did not observe statistically significant correlations with the Repeatable Battery for the Assessment of Neuropsychological Status (RBANS) attention subscale for the full sample—even though CMI patients scored significantly lower than controls on the attention subscale. However, controls’ FA had a significant positive correlation with coding performance when analysed separately, although no such correlation was present in the CMI data. Houston et al. also observed that FA was positively correlated with self-reported pain (i.e., McGill Pain Questionnaire-short form) scores—and that group differences in FA were eliminated when pain was statistically controlled, suggesting that pain accounted for group differences in FA. Consequently, Kumar et al.^9^ and Houston et al.^8^ came to different conclusions regarding correlations between FA and cognitive performance. Kumar observed significant correlations between cognitive performance and FA—but Houston et al.^8^ did not, except among controls. Indeed, Houston et al.^8^ found that pain levels rather than cognitive performance were correlated with FA levels in different regions of interest (ROIs).

However, it remains unclear whether (i) CMI is associated with differences in functional connectivity between fibre tracts of the cerebellum and cerebrum,^16–18^ and (ii) whether differences in functional connectivity are associated with cognitive dysfunction and/or the chronic pain experience of CMI patients. It is hypothesized that differences in rs-fMRI indices will be observed between CMI patients and healthy matched controls. Furthermore, where CMI patients show lower seed-to-voxel functional connectivity between ROIs than controls (i.e. relative hypoconnectivity) it will be representative of a transmission deficit that will be reflected by poorer attention scores on a standardized assessment. However, where CMI patients show greater seed-to-voxel functional connectivity between ROIs than controls (i.e. relative hyperconnectivity), it will represent activity representative of chronic pain inhibition and be associated with self-reported chronic pain levels.

## Materials and methods

### Participants

Eighteen CMI patients and 18 controls matched for age, sex and education participated in this study. CMI patients were diagnosed by either MGL or SV based upon radiological evidence and symptom profile and were recruited during presurgical consultation. Healthy controls were recruited from the Akron and Cleveland community. No participants reported using opioids for pain management. Participants were compensated $100 for their time and were permitted a copy of their scanned images.

### Procedure

Participants completed MRI scans and a neuropsychological assessment, typically within one week. This study was approved by local institutional review boards of the University of Akron and the Cleveland Clinic, and written informed consent was provided by all participants. Imaging for all CMI patients occurred prior to undergoing surgery and after undergoing presurgical consultation. The RBANS was administered prior to surgery for 16 CMI patients and following surgery for two CMI patients. For the two who were assessed post-surgery, assessment was conducted approximately three months later to avoid post-surgery complications and surgery-related pain. Outlier analysis and visual inspection of the data from these two participants indicated that their data did not meaningfully differ from that of the 16 patients. Thus, results were interpreted for the full sample of 18 CMI patients and their matched controls.

### Self-report and cognitive measures

Participants completed the short-form McGill Pain Questionnaire as a self-report measure of chronic pain, consisting of 15 questions where subjects are asked to rate their pain on a Likert-type scale (0 = none to 5 = worst possible). The Chiari Symptom Profile (CSP) was also administered to participants. The CSP is comprised of 57 items on a 0–4 scale used to assess aspects of Chiari Malformation and treatment outcomes, where higher scores indicate greater disability.

The RBANS has been used to assess cognitive status for a variety of acquired, progressive neurological and neuropsychiatric conditions.^19–21^ The attention subscale was used as a covariate in this study (see Table 1). The attention subscale consists of the digit symbol coding task, often considered a measure of processing speed, and the forward digit span, commonly thought to reflect working memory.

**Table 1:** Demographic and clinical characteristics by CMI status

Measures	CMI	Control	*P*
No. of participants	17 (1 male)	18 (1 male)	–
Age, years	32.5 (10.1)	37.3 (14.3)	0.262^*^
Years of education	14.2 (2.4)	14.2 (2.2)	0.865
Tonsillar position	**12.6 (4.8)**	**1.4 (1.9)**	**<0.001**
No. with syrinx	4	0	–
MPQ pain	**26.0 (21.3)**	**6.1 (11.7)**	**<0.001**
CSP—Headaches	**2.8 (1.0)**	**1.2 (0.7)**	**<0.001**
CSP—Neck pain	**2.8 (1.4)**	**0.7 (0.7)**	**<0.001**
CSP—Arm pain	**2.2 (1.5)**	**0.4 (0.9)**	**<0.001**
CSP—Back pain	**2.5 (1.3)**	**1.2 (1.0)**	**0.005**
CSP—Dizziness	**1.8 (1.1)**	**0.6 (0.6)**	**<0.001**
CSP—Tinnitus	**1.8 (1.5)**	**0.4 (0.8)**	**0.004**
CSP—Difficulty concentrating	**2.2 (1.3)**	**0.9 (0.8)**	**0.003**
CSP—Insomnia	**2.2 (1.3)**	**0.8 (1.1)**	**0.001**
CSP—Chronic fatigue	**2.7 (1.4)**	**1.1 (0.9)**	**0.001**
CSP—Irritability	**2.1 (1.1)**	**0.9 (0.8)**	**0.002**
CSP—Forgetfulness	**2.0 (1.1)**	**0.4 (0.7)**	**<0.001**
CSP—Head pain	**2.3 (1.4)**	**0.1 (0.4)**	**<0.001**
CSP—Generalized body pain	**2.1 (1.7)**	**0.3 (0.7)**	**<0.001**
DASS total	**19.4 (13.0)**	**8.6 (9.8)**	**0.008**
RBANS Attention subscale	**90.9 (21.5)**	**114.0 (19.4)**	0.002*

Values in parentheses indicate standard deviations by group. Bolded values represent differences in measurement or scale score derived from the Wilcoxon rank-sum test. CMI, type I Chiari malformation; CSP, Chiari Symptom Profile (scale values correspond to the following: 0 = ‘never’, 1 = ‘rarely’, 2 = ‘some of the time’, 3 = ‘most of the time’, 4 = ‘all of the time’); DASS, Depression, Anxiety, and Stress Scale; MPQ, short-form McGill Pain Questionnaire; RBANS, Repeatable Battery for the Assessment of Neuropsychological Status (attention subscale comprised of digit span and digit coding scores).

* *P* values derived from a Student’s *t*-test.

### MRI data acquisition

Imaging was performed on a 3 tesla Siemens Prisma with a standard 20 channel head-neck array (Siemens Healthineers, Erlangen, Germany) at the Cleveland Clinic. For the rs-fMRI protocol, participants were instructed to lie still with eyes closed during the blood oxygenation level-dependent (BOLD) scan (single-shot echo planar imaging, axial, 256 mm × 256 mm FOV, 128 × 128 matrix, 30 4 mm thick slices with no gap, time to echo/repetition time = 28/2800 msec, flip angle 80°, 137 measurements).

### Seed-to-voxel analysis

Seed-based correlational analyses were performed to evaluate group-differences in resting-state blood oxygenation level-dependent activation using the Functional Connectivity (CONN) toolbox (www.nitrc.org/projects/conn, RRID: SCR_009550) with statistical parametric mapping 12 (http://www.fil.ion.ucl.ac.uk/spm; Wellcome Trust Centre for Neuroimaging, University College London, UK) in MATLAB (R2019b). For this study, functional connectivity differences were examined using the following seeds: (i) the frontoparietal pathway, given prior literature demonstrating a deficit in self-focused attention in CMI patients^13^; (ii) the posterior cingulate cortex (PCC), given its association with the default mode network and the connection between CMI and experience of depression and pain^13^^,^^22^; (iii) the posterior cerebellar pathway (PCP), due to the structural damage to the cerebellum in CMI and the link the PCP has with other brain regions supporting higher order cognition^23^; and (iv) Crus I of the cerebellum given the implication of this region in higher order executive functions and the default mode network.^16^^,^^17^ Given the structural differences in the cerebellum of CMI patients,^24^ seeds in the vermis and lobules of the cerebellum were also used.

All steps were completed using the CONN toolbox. Default preprocessing of the functional data included discarding the first three image volumes to allow for T1 equilibrium and to limit movement artefacts. Images were also realigned to estimate and correct for subject head motion and underwent slice-timing correction for differences in slice time acquisition between scans. Structural scans were segmented into cerebrospinal fluid, grey matter and white matter to remove confounding signals. Maps of functional connectivity were then co-registered to the structural sequences. Subsequently, images were normalized to Montreal Neurological Institute normalization space and were smoothed using an 8 mm Gaussian Kernel. Intermediate (i.e., 97th percentile) artefact detection tool (ART) outlier detection was used to identify outlier and tissue confounds, which were added to the model as first level covariates and were subsequently removed using linear-regression and bandpass-filtering (0.01–0.1). Through the CONN toolbox, severe imaging artefacts were identified in one CMI patient’s images, rendering their data unusable.

First-level seed-to-voxel analysis was computed using general linear model, which computes the correlation from a seed ROI with all other brain voxels for each seed within each individual. Seeds were selected from the list of pre-defined ROIs within CONN, and included the frontoparietal pathway, PPC, PCP, Crus I, lobule III, lobule VIII, Vermis I and II, Vermis VII, and Vermis IX. Three factors drove the selection of these seeds. First, the anterior cingulate cortex has been associated with pain processing—and a central goal of this study was to determine whether CMI was associated with the functional connectivity involving this region. Second, given the apparent attention deficits in CMI,^4^ it was of interest to examine the functional connectivity of structures involved in the frontoparietal attentional pathway.^25^ Third, one prediction of the Schmahmann hypothesis of cognitive processing in the cerebellum^18^ is that tissue damage in the cerebellum can cause cognitive deficits.^26^ This is the rationale for using cerebellar seeds and following these pathways. Consequently, we assessed the functional connectivity of structures associated with the cortico-ponto-cerebellar pathway from the prefrontal cortex through the pons and middle cerebellar peduncle to the cerebellum as well as structures along the cerebello-thalamo-cortical pathway from the cerebellum through the superior cerebellar peduncle via the thalamus to the prefrontal cortex.^27^

### Statistical analysis

Within-group correlations underwent whole brain false discovery rate (FDR) correction to maintain an alpha of <0.05. Second-level analyses controlling for attention and pain scores were conducted to examine differences between-group statistical parametric maps. FDR was controlled for in the group comparisons using a voxel-level height threshold of *P* < 0.05 and cluster-level extent threshold (pFDR-corr < 0.05—Whitfield-Gabrieli and Nieto-Castanon^28^).

### Data availability

The data that support the findings of this study are available from the corresponding author, upon reasonable request.

## Results

**Figure 1:**
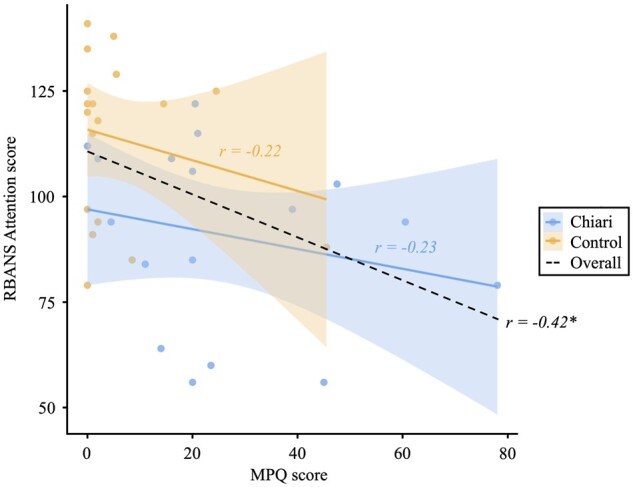
Associations between attention performance and self-reported pain.

Seventeen adult CMI patients and 18 matched controls comprised the final sample. Demographic and clinical information is provided in Table 1. Of interest, patients and controls were well-matched for age (*M*_CMI_ = 32.5 years, *M*_control_ = 37.3 years) and years of education (*M*_CMI_ = 14.2 years, *M*_control_ = 14.2 years). CSP outcomes indicated that CMI patients presented with typical symptomology. As reported previously,^5^ CMI patients exhibited significantly worse performance on the RBANS attention scale and reported significantly more pain than healthy controls (both *P *<* *0.003). Additionally, attention performance was correlated with chronic pain across all participants, *r*(33) = −0.42, *P = *0.002, but not separately within each group (see Fig. 1).

**Figure 2:**
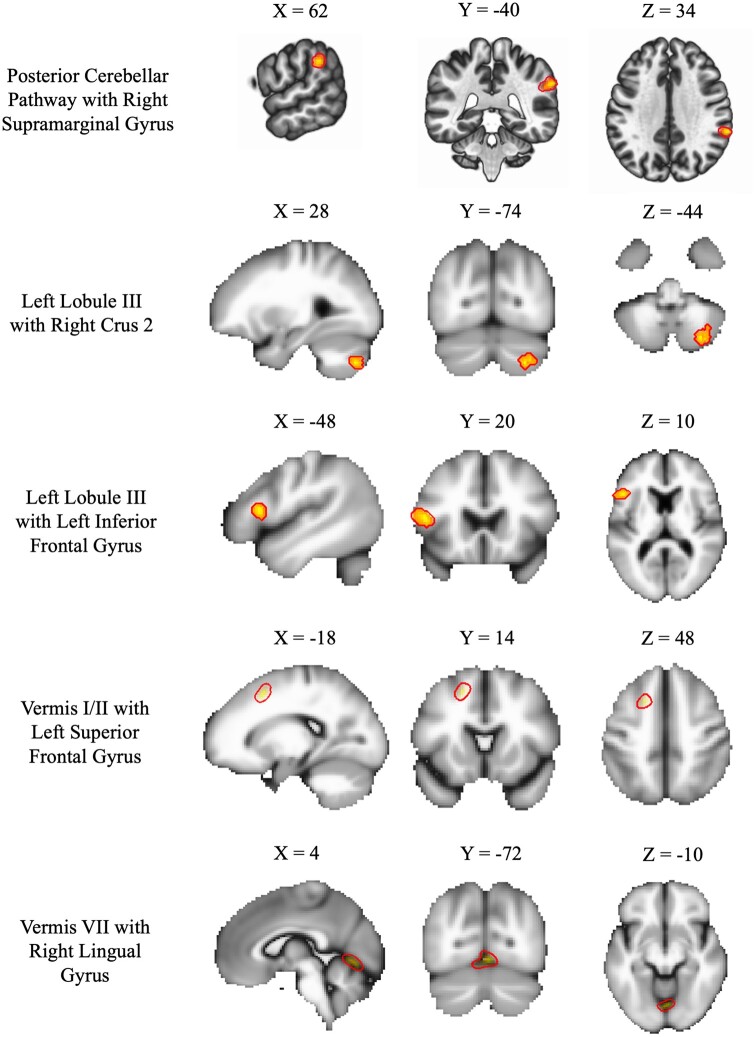
Significant positive contrasts between seeds and areas of connectivity.

Between-group comparisons demonstrated different patterns of functional connectivity between patients and controls (see Table 2). Specifically, CMI patients showed higher functional connectivity between the PCC and the left globus pallidus, *t*(33) = −5.95, *P* < 0.001, and left parahippocampal gyrus, *t*(33) = −5.95, *P* < .001. Additionally, there was a pattern of hyperconnectivity for patients compared to controls between Crus I and the left superior frontal gyrus (SFG), *t*(33) = −5.33, *P* < .001. Hyperconnectivity in patients was also found between cerebellar lobule VIII with part of the left postcentral gyrus, *t*(33) = −6.2, *P* < 0.001, and Vermis IX and the precuneus, *t*(33) = −4.74, *P* < 0.001 (see Fig. 2).

**Table 2:** Significant group differences in functional connectivity.

Seed	Contrast	Region of interest	MNI coordinates (*X*, *Y*, *Z*)	Beta	Peak *T*-value	*p*-FDR	Cluster size (Voxels)*
Posterior cingulate cortex	CMI > Controls	Left globus pallidus	(−26, −10, −02)	−0.19	−5.95	<0.001	198
		Left parahippocampal gyrus	(−16, −36, −34)	−0.19	−5.51	<0.001	166
	Controls > CMI	*–*	*–*	–	–	–	–
Posterior cerebellar pathway	CMI > Controls	*–*	–	–	–	–	–
	Controls > CMI	Right supramarginal gyrus	(62, −40, 34)	0.29	4.66	<0.001	248
Crus I	CMI > Controls	Left superior frontal gyrus	(−16, 12, 58)	−0.21	−5.33	<0.001	169
	Controls > CMI	–	–	–	–	–	–
Left lobule III	CMI > Controls	–	–	–	–	–	–
	Controls > CMI	Left inferior frontal gyrus	(−48, 20, 10)	0.18	5.29	< 0.001	144
		Right Crus 2	(28, −74, −44)	0.19	5.20	< 0.001	149
Left lobule VIII	CMI > Controls	Left postcentral gyrus	(−38, −34, 46)	−0.18	−6.20	< 0.001	617
	Controls > CMI	–	–	–	–	–	–
Vermis I and II	CMI > Controls	*–*	–	–	–	–	–
	Controls > CMI	Left superior frontal gyrus	(−18, 14, 48)	0.18	5.08	< 0.001	145
Vermis VII	CMI > Controls	–	*–*	–	–	–	–
	Controls > CMI	Right lingual gyrus	(4, −72, −10)	0.28	5.32	<0.001	250
Vermis IX	CMI > Controls	Precuneus	(0, −64, 46)	−0.25	−4.74	<0.001	272
	Controls > CMI	–	–	–	–	–	–
Frontoparietal attentional pathway	CMI > Controls	–	–	–	–	–	–
	Controls > CMI	–	–	–	–	–	–

*p*-FDR—comparison probability values after false discovery rate correction *Voxel size = 2 × 2 × 2 mm.

**Figure 3:**
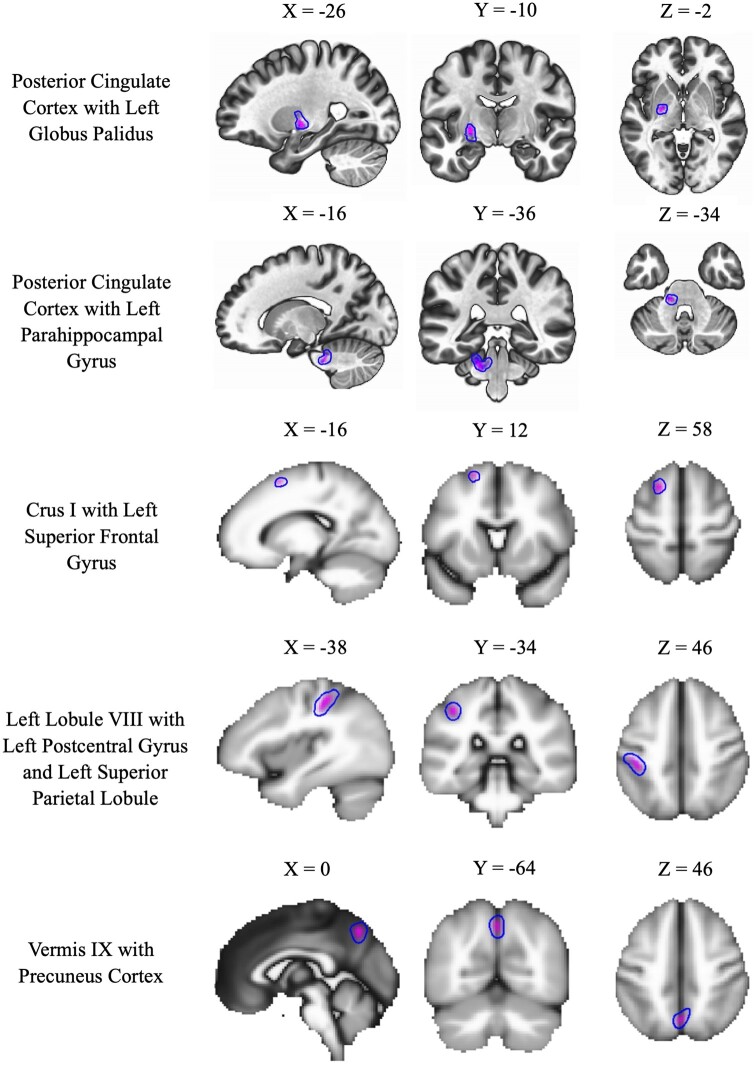
Significant negative contrasts between seeds and areas of connectivity.

There was also a pattern of hypoconnectivity in CMI patients between the PCP seed and the right supramarginal gyrus compared to healthy controls, *t*(33) = 4.94, *P* < .001. Within the cerebellum, there was hypoconnectivity between the left lobule III and the left inferior frontal gyrus, *t*(33) = 5.29, *P* < 0.001, and the right Crus II, *t*(33) = 5.20, *P* < .001. Further hypoconnectivity was observed between Vermis I/II and the left SFG, *t*(33) = 5.08, *P* < 0.001 and between Vermis VII and the right lingual gyrus (LG), *t*(33) = 5.32, *P* < 0.001 (see Fig. 3). There were no differences in functional connectivity using any area of the frontoparietal pathway as a seed.

To determine whether differences in functional connectivity could be associated with CMI-related deficits in attention performance, RBANS attention scores were added to the general linear model model as a covariate. With attention scores added to the model, CMI patients no longer exhibited a pattern of hyperconnectivity between the PCC and both the left parahippocampal gyrus and left globus pallidus, and between the Crus I and left SFG. However, hyperconnectivity between left Lobule VIII and the left postcentral gyrus remained significant, *t*(32) = −5.86, *P* < 0.001, as well as between Vermis IX and the precuneus, *t*(32) = −4.21, *P* < .001. Additionally, the pattern of hypoconnectivity between the PCP and right supramarginal gyrus remained significant after controlling for attention performance, *t*(32) = 4.50, *P* < .001. All other patterns of hypoconnectivity were no longer significant after controlling for attention scores. Results for group differences in patterns of activation after controlling for RBANS attention scores are summarized in Table 3.

**Table 3:** Significant group differences in functional connectivity after controlling for attention

Seed	Contrast	Region of interest	MNI coordinates (*X*, *Y*, *Z*)	Beta	Peak *T*-value	*p*-FDR	Cluster size (Voxels)*
Posterior cingulate cortex	CMI > Controls	–	–	–	–	–	–
Posterior cerebellar pathway	Controls > CMI	Right supramarginal gyrus	(-62, -40, 32)	0.28	4.5	<0.001	190
Crus I	CMI > Controls	–	–	–	–	–	–
Left lobule III	Controls > CMI	–	–	–	–	–	–
Left lobule VIII	CMI > Controls	Left postcentral gyrus	(−38, −34, 46)	−0.21	−5.86	<0.001	341
Vermis I and II	Controls > CMI	–	–	–	–	–	–
Vermis VII	Controls > CMI	–	–	–	–	–	–
Vermis IX	CMI > Controls	Precuneus	(0, −64, 46)	−0.27	−4.21	<0.001	121

*p*-FDR—comparison probability values after false discovery rate correction

* Voxel size = 2 × 2 × 2 mm.

As with attention performance, a separate general linear model model was created that controlled for the greater pain scores reported by CMI patients. After controlling for McGill pain scores, there was no longer evidence of CMI-related hyperconnectivity between the PCC and the left parahippocampal gyrus. Yet, hyperconnectivity remained in the left globus pallidus, *t*(32) = −5.43, *P* < .001. CMI patients also still displayed relative hyperconnectivity between Vermis IX and the precuneus, *t*(32) = −4.71, *P* < .001. Finally, patterns of hypoconnectivity remained significant after controlling for pain between the PCP and right supramarginal gyrus, *t*(32) = 4.66, *P* < 0.001, as well as Vermis VII and the right LG, *t*(32) = 5.32, *P* < .001. No other patterns of hypoconnectivity were apparent after controlling for pain (see Table 4).

**Table 4:** Significant group differences in functional connectivity after controlling for pain

Seed	Contrast	Region of interest	MNI coordinates (*X*, *Y*, *Z*)	Beta	Peak *T*-value	*p*-FDR	Cluster size (Voxels)*
Posterior cingulate cortex	CMI > Control	Left globus pallidus	(−26, −10, −02)	−0.21	−5.43	<0.001	173
Posterior cerebellar pathway	Control > CMI	Right supramarginal gyrus	(62, −40, 34)	0.29	4.66	<0.001	248
Crus I	CMI > Control	–	–	–	–	–	–
Left lobule III	Controls > CMI	–	–	–	–	–	–
Left lobule VIII	CMI > Controls	Left postcentral gyrus	(−40, −26, 40)	−0.22	−8.48	<0.001	281
Vermis I and II	Controls > CMI	–	–	–	–	–	–
Vermis VII	Controls > CMI	Right lingual gyrus	(2, −74, −12)	0.34	5.32	<0.001	329
Vermis IX	CMI > Controls	Precuneus	(0, −64, 46)	−0.29	−4.71	<0.001	281

*p*-FDR—comparison probability values after false discovery rate correction *Voxel size = 2 × 2 × 2 mm.

## Discussion

### Interpretation of results

Prior research has demonstrated microstructural differences in CMI patients compared to controls using DTI methods, but it remained unexplored whether those differences were accompanied by deficits in functional connectivity—especially in brain areas with known connections to the cerebellum. The present results provide evidence for functional connectivity differences in CMI patients and age- and education-matched controls using a prospective design. Specifically, two primary patterns of results were found: pathways where CMI patients showed greater functional connectivity, and those where CMI showed lesser connectivity. Additionally, there was partial support for the study hypotheses. The hypothesis that hypoconnectivity would be associated with attention subscales of the RBANS was confirmed for all seeds with the exception of the PCP. That is, with the exception of the PCP, controlling for the effect of attention eliminated all hypoconnectivity differences between CMI patients and controls. Furthermore, the hypothesis that hyperconnectivity would be associated with pain was supported with the exception of the connectivity between the PCC and left globus pallidus, left lobule VIII and left postcentral gyrus and Vermis IX and precuneus. However, there was also evidence that hyperconnectivity was generally associated with pain and that hypoconnectivity was associated with attention. While not directly in line with the hypotheses, attention and pain are closely related, with pain drawing upon available cognitive resources (e.g. attention), thereby likely leading to deficits in these functions. Implications of these findings are discussed below.

Beginning with the finding of CMI relative hyperconnectivity in the PCC, it is important to note the role of the posterior cingulate as a central hub for the default mode network. Disruption of default mode dynamics have been identified in chronic pain patients.^22^^,^^29^ Thus, the relative hyperconnectivity exhibited by CMI patients may provide further evidence of the chronic pain experienced by CMI patients. Moreover, these findings also inform the hypothesis that many of the cognitive deficits manifested in CMI stem from the distracting effects of pain.^5^^,^^6^^,^^11^ Additionally, greater connectivity between Crus I and the left SFG, left lobule VIII and the left postcentral gyrus, and Vermis IX and the precuneus also support a pattern of altered cognitive processing due to the effect of pain requiring greater upregulation of inhibitory control to maintain focus.^30^ Importantly, because the pattern of relative hyperconnectivity for the PCC left lobule VIII and Vermis IX seeds persisted after controlling for self-reported pain, this also supports the findings of altered white matter morphometry in CMI.^8^^,^^9^

There was also evidence of relative hypoconnectivity between the PCC and the left parahippocampal gyrus, left lobule III and the left inferior frontal gyrus and right Crus 2, Vermis I/II and the left SFG, and Vermis VII and the right LG in the CMI patients. Many of these differences were eliminated once covarying for pain and attention, suggesting a relationship between the hypoconnectivity of these pathways, cognitive dysfunction, and pain. However, other relationships, including those relationships involving Vermis VII and the PCP, remained. These results are consistent with Schmahmann’s hypothesis of tissue damage in the cerebellum in CMI resulting in functional connectivity deficits.^31^^,^^32^ Specifically, because this functional hypoconnectivity between the PCP and right supramarginal gyrus remained significant after controlling for group differences in pain and attention, it seems unlikely that these group differences are associated with cognitive dysfunction or pain in CMI. It is also relevant that the supramarginal gyrus is implicated in visuospatial reasoning and spatial orientation. Prior literature has demonstrated that CMI patients do not perform as well as their healthy counterparts on such measures.^5^ Thus, deficits in functional connectivity between the PCP and the supramarginal gyrus may still provide a partial explanation for these previously observed differences in cognitive function and will benefit from further investigation. Furthermore, the persistence of the relative hypoconnectivity between Vermis VII and the right LG after controlling for pain is also of interest. While speculative, the sensitivity of this finding for attention, but not pain, suggests that the corresponding pathway is uniquely associated with attention dysfunction in CMI and at least partially independent of the CMI pain experience.

It was also found that CMI patients had relative hyperconnectivity between left lobule VIII and a large cluster comprised of the left postcentral gyrus. Similar to the anterior lobes of the cerebellum (i.e. lobules I–V), lobule VIII is associated with sensorimotor function.^33^^,^^34^ Increased activation between lobule VIII and the postcentral gyrus in patients is interesting, given the involvement of these areas in spatial processing and visuomotor perception.^35^ It has also been suggested that functional connectivity in this region is associated with chronic pain. For example, Kong et al.^36^ found that individuals with high levels of pain exhibited greater activation in the postcentral gyrus. Yet, in this study, this regional group difference remained significant after controlling for pain and attention. It is feasible that this relative connectivity difference resulted from a change in the functional structure due to prolonged sensations of pain (e.g. central sensitization). However, further work is needed to reconcile the independence of the CMI-related functional connectivity differences from cognitive dysfunction and chronic pain covariates.

### Vermian role in CMI cognitive regulation

Among the several novel findings in this study, the functional connectivity differences involving the cerebellar vermis are particularly interesting. The vermis, while traditionally associated with regulation of posture and movement, has also been shown to be associated with feelings of pain and anxiety, and disruptions to the cerebro-cerebellar connections from the vermis have been shown to be associated with emotional dysfunction.^33^^,^^37^ Within the posterior vermis, Vermis IX has been associated with cognitive and emotional dysfunction through cerebellar–limbic pathways.^33^ Additionally, the pattern whereby patients demonstrated heightened connectivity between Vermis IX and the precuneus, which are associated with affective responses to pain and self-consciousness, is in line with prior evidence suggesting there is a pain distractor effect in CMI patients,^13^ likely leading to downstream cognitive deficits. It could be that long-term pain processing has led to a functional alteration of this cerebellar-limbic pathway (e.g. a neuropathic effect^8^). Yet, the observed group differences were independent of pain, emphasizing the undiscovered complexities of the relationship between this pathway, cognitive dysfunction, and the pain experience. Additionally, Vermis VII is associated with higher order cognitive functions.^33^ The pattern of under-activation in patients between the Vermis VII and the right LG, which is associated with visuospatial reasoning, is also in line with evidence demonstrating a deficit in visuospatial reasoning in CMI patients.^5^ As previously discussed, this finding remained after controlling for the effect of pain, but not attention.

Connectivity between Vermis I/II and the left SFG was also reduced in patients compared to controls, but this pattern was eliminated after controlling for both attention and pain separately. The role of the Vermis I/II is not entirely understood, although there is some agreement that the anterior region of the cerebellum (lobules I–V) tend to play a larger role in motoric function than cognitive function, meanwhile the posterior region (lobules VI–X) are more involved in cognitive processes.^17^^,^^23^^,^^33^ Furthermore, given the small area and variability in size of the anterior vermis, Schmahmann et al.^38^ recommend referring to this collection of structures as a vermal area. Thus, integrating the results of Vermis I/II with those of hemispheric lobule III may provide a clearer case. Hypoconnectivity was also identified between the lobule III seed and the left inferior frontal gyrus and the right Crus II. Together, these findings are potentially important for understanding the effect of CMI on cognition given the association that the inferior and superior gyri have with higher order cognitive processes. Specifically, the inferior frontal gyrus is implicated in response inhibition^39^ and language processing^40^; meanwhile, the left SFG is implicated in working memory^41^ and response inhibition.^42^ However, Liakakis et al.^40^ found a cluster within the left inferior frontal gyrus that was associated with viewing fine motor movements, which the authors interpreted as demonstrating activation of the mirror neuron system. Integrating these findings with the understanding that the anterior cerebellum is associated with motor movement, it could be that the observed pattern of hypoconnectivity between these regions is associated with reduced fine motor control in CMI patients. Another explanation could be that there are projections stemming from the anterior cerebellum to the prefrontal cortex that are associated with emotion.^43^ Indeed, this explanation is more in line with the present findings, given that when pain and attention were included, these differences were eliminated.

### Limitations

Although this study largely establishes the literature on resting-state functional connectivity differences in CMI, it is not without limitation. First, given the relatively small sample size, the design of this study is limited in its ability to infer directional correlations between self-reported pain values and patterns of rs-fMRI activation. Second, although we emphasized a matched case–control design, our CMI sample had relatively high levels of education, which may not reflect that of the entire CMI population. Finally, the use of seed-based analyses requires that these seeds be selected *a priori*, limiting findings to the selected seeds of interest.^44^ Alternatively, voxel-to-voxel independent component analysis could have been conducted to examine patterns of brain connectivity free of *a priori* constraints. However, the independent component analysis approach is unable to provide insight into network-to-network communication and relies on a pre-specified number of components such that a network could be broken into subnetworks and not accurately reflected as a singular component.^44^ Moreover, the choice to conduct solely seed-based analyses in this study was made in part to minimize the likelihood of false discovery. In other words, because seed-based and independent component analysis analysis approaches can yield dissimilar results,^45^^,^^46^ it was determined that a seed-based approach targeting regions previously indicated as potential mechanisms for CMI-related cognitive and emotional dysfunction would provide the optimal approach to detect potential effects and minimize the likelihood of Type I error.

## Conclusion

The goal of this study was to determine whether there were functional connectivity differences between CMI patients and healthy controls as have been shown for structural connectivity.^8^ Patterns of both hyper- and hypoconnectivity were identified between CMI patients and matched healthy controls. Patterns of connectivity suggest that pain and attention draw upon neural resources, likely resulting in downstream deficits in cognition. Additionally, pathways of hypoconnectivity were identified that were independent of pain and attention, which are posited to be the result of functional alteration of the pathway due to prolonged pain sensation. Further work is needed to clarify the mechanisms of CMI-related abnormalities in resting functional connectivity and whether these abnormalities can serve as an informing factor for treatment outcomes. Future research should also continue to piece together the structural (both macro and micro), functional, and behavioural features of Chiari Malformation and the implications of cerebellar structure and connectivity on cognitive function and the pain experience.

## Funding

The authors thank the Conquer Chiari Foundation (https://www.conquerchiari.org) for contributing funding to this study. 

## Competing interests

The authors report no competing interests. 

## Abbreviations

CMI =: Type I Chiari malformationCONN = name of functional connectivity analysis toolbox
CSP =: Chari Symptom Profile
DTI =: diffusion tensor imaging
FA =: fractional anisotropy as identified by diffusion tensor imaging
FDR =: false discovery rate
LG =: lingual gyrus
MNI =: Montreal Neurological Institute normalization
PCC =: posterior cingulate cortex
PCP =: posterior cerebellar pathway
RBANS =: Repeatable Battery for the Assessment of Neuropsychological Status
ROI =: region of interest
rs-fMRI =: resting-state functional MRI
SFG =: superior frontal gyrus
